# Symbiont diversity within *Loripes orbiculatus* and the case for multiple hosts

**DOI:** 10.1093/ismejo/wrag094

**Published:** 2026-04-15

**Authors:** Margaret A Vogel, Fragkiskos Machairas, Sophia Ferchiou, Jay Osvatic, Hanin Alzubaidy, Joana Séneca, Bela Hausmann, Katja Klun, Jillian M Petersen

**Affiliations:** Department of Fundamental Microbiology, University of Lausanne, Lausanne, 1015 Vaud, Switzerland; Center for Microbiology and Environmental Systems Science, University of Vienna, 1030 Vienna, Austria; Center for Microbiology and Environmental Systems Science, University of Vienna, 1030 Vienna, Austria; Center for Microbiology and Environmental Systems Science, University of Vienna, 1030 Vienna, Austria; Joint Microbiome Facility of the Medical University of Vienna and the University of Vienna, University of Vienna, 1030 Vienna, Austria; Division of Clinical Microbiology, Department of Laboratory Medicine, Medical University of Vienna, 1090 Vienna, Austria; GEOMAR, Helmholtz Center for Ocean Research, 24148 Kiel, Germany; Center for Microbiology and Environmental Systems Science, University of Vienna, 1030 Vienna, Austria; Joint Microbiome Facility of the Medical University of Vienna and the University of Vienna, University of Vienna, 1030 Vienna, Austria; Joint Microbiome Facility of the Medical University of Vienna and the University of Vienna, University of Vienna, 1030 Vienna, Austria; Division of Clinical Microbiology, Department of Laboratory Medicine, Medical University of Vienna, 1090 Vienna, Austria; Marine Biology Station Piran, National Institute of Biology, 6330 Piran, Slovenia; Center for Microbiology and Environmental Systems Science, University of Vienna, 1030 Vienna, Austria; Vienna Doctoral School in Microbiology and Environmental Science, University of Vienna, 1030 Vienna, Austria; Environment and Climate Research Hub, University of Vienna, 1090 Vienna, Austria

**Keywords:** lucinid bivalves, seagrass, symbiosis, *Ca.* Thiodiazotropha, facilitation

## Abstract

Seagrasses support immense biodiversity and are critical for maintaining coastal ecosystem health. These foundation species benefit from a “three-way” facultative relationship with one of the common inhabitants of seagrass meadows, lucinid bivalves, which host specific bacterial *Candidatus* Thiodiazotropha symbionts. Relatives of the bivalve symbionts have been detected on seagrass roots, raising the possibility that these symbionts may colonize both animals and plants; however, no study has yet compared bivalve- and seagrass-associated symbionts at the same site and time. Our combination of 16S ribosomal RNA (rRNA) gene amplicon and metagenome sequencing revealed a greater diversity than was previously observed within both lucinid bivalves and on seagrass roots from the Adriatic Sea and resulted in the closed genome of one prominent symbiont species. We show that two of the *Ca.* Thiodiazotropha ASVs found on seagrass roots are identical to those found in bivalve hosts at the same site. This suggests that symbiont sharing may occur in the seagrass habitat between these two host species, which has important evolutionary and ecological implications for both hosts and symbionts.

## Introduction

The immense biodiversity found in seagrass meadows worldwide is supported in part by the dense habitat matrix created by seagrasses. These foundation species also provide valuable ecosystem services, including stabilizing sediments, oxygenating the water column, and blue carbon sequestration, making seagrasses critical for maintaining healthy coastal ecosystems. These highly productive systems also generate high rates of organic matter decomposition, which can lead to increased and potentially phytotoxic levels of sulfide in the sediment [1]. Although seagrasses have evolved their own mechanisms to mitigate sulfide toxicity [2], they also rely on facilitative interactions with their inhabitants, including lucinid bivalves [3].

*Lucinidae* is a species-rich family of bivalves characterized by their obligate symbiosis with sulfur-oxidizing Gammaproteobacteria that are housed within specialized cells called bacteriocytes in the gill tissue. These endosymbionts oxidize reduced sulfur and form a nutritional symbiosis with the bivalve host by delivering organic carbon. Lucinid bivalves have a global distribution and commonly occur within seagrass meadows [4, 5]. Diversification of lucinids occurred during the Cretaceous period, concurrent with the emergence and diversification of seagrasses [6]. The chemical cycling performed by the symbionts in lucinid gills lowers sulfide levels in the sediment, which can, in turn, benefit the surrounding seagrass and facilitate growth [4]. Not only has the presence of lucinids been shown to increase seagrass growth and survival under stressful conditions [3, 7], but members of the genus *Candidatus* Thiodiazotropha, the group to which many lucinid symbionts belong, have also been found to be commonly associated with the roots of many seagrass species [8].

Recent phylogenomic and comparative genomic analyses have expanded our understanding of the ecology and evolutionary history of *Ca.* Thiodiazotropha symbionts. Species of *Ca.* Thiodiazotropha are found associated with hosts not only in shallow water habitats but also in the deep-sea near hydrothermal vents and cold seeps where symbiont switching by the host has been observed [5, 9]. Metagenome-assembled genomes (MAGs) have shown that species belonging to *Ca.* Thiodiazotropha share the same core metabolic capabilities; however, these species exhibit diverse genotypes including nitrogen fixation limited to shallow water species [9]. In shallow water habitats, these symbionts are acquired by lucinids during the early stages of host development from the environment, where they occur in low abundances [8, 10, 11]. A single species belonging to *Ca.* Thiodiazotropha can have associations with a diverse range of lucinid host species. Additionally, multiple *Ca.* Thiodiazotropha species have been shown to associate with the same lucinid species and can even occur within the same host individual. For example, two *Ca.* Thiodiazotropha species were observed within the gill tissue but separate bacteriocytes of *Cathrolucina costata* [5]. This flexibility in the association *Ca.* Thiodiazotropha and their lucinid hosts could be fundamental to their widespread distribution and the evolutionary success of this symbiosis.

Although *Ca.* Thiodiazotropha has been studied separately in association with either marine plants or lucinid bivalves, no study has investigated its symbioses with both hosts where they co-occur (e.g. [5, 9, 12–14]). Here, we report a fine-scale investigation of *Ca.* Thiodiazotropha diversity within 86 bivalve host individuals and several compartments of the surrounding seagrass environment at two sites in the Adriatic Sea. A combination of 16S ribosomal RNA (rRNA) gene amplicon and metagenome sequencing revealed a greater diversity than was previously observed within the bivalve gills, as well as on seagrass rhizomes and roots. Additionally, this shows that select *Ca.* Thiodiazotropha ASVs found on seagrass roots and rhizomes are identical to those found in their bivalve host, *Loripes orbiculatus,* at the same site. This finding extends beyond previous research showing the relatedness of seagrass and bivalve symbionts by suggesting that some of these symbionts may be shared between these two hosts. Thus, seagrasses could act as an additional host for this genus of symbionts, or lucinid bivalves could act as an additional host for important symbionts of seagrasses.

## Materials and methods

### Site description

**Figure 1:**
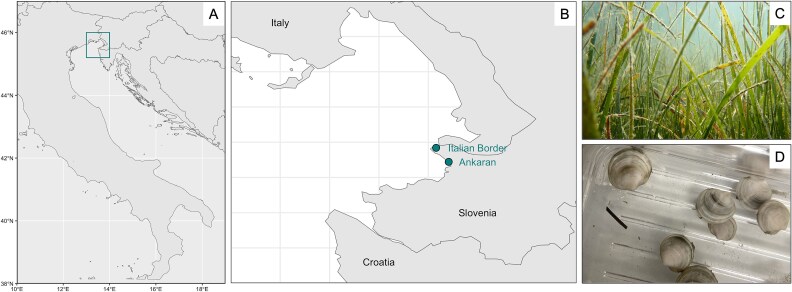
Location of the field sites and host organisms. (A) Map of the location of the field sites off the coast of Slovenia in the Adriatic Sea, (B) detailed map of the Ankaran and Italian Border sites, (C) the submerged seagrass bed at Ankaran, and (D) *L. orbiculatus* taken from the field sites.

All samples were collected off the coast of Slovenia in the Gulf of Trieste at two nearby sites (Fig. 1): Italian Border (IB; 45.592025°N, 13.715745°E) and Ankaran (AK; 45.572113°N, 13.741289°E). Both sites are characterized by seagrass habitats with *Cymodocea nodosa* (commonly known as little Neptune grass) as the dominant seagrass species. The Ankaran site also features a second distinct intertidal seagrass bed composed of *Zostera noltii* (commonly known as dwarf eelgrass), which becomes exposed at low tide.

### Lucinid sampling

To characterize the symbiont diversity at the two sites, *L. orbiculatus* individuals were collected from August to October 2021 from both sites (AK and IB). Sediment from in and around the seagrass beds at each site was collected into buckets and topped with seawater for transport back to the laboratory at the National Institute of Biology (NIB) Marine Biology Station Piran. Upon returning to the laboratory, sediments were sieved to isolate *L. orbiculatus.* The individuals collected for metagenome sequencing were also used in a separate experiment. All individuals were dissected, and both gills were placed whole in RNAlater and frozen at −80°C. An additional collection of *L. orbiculatus* individuals took place in March 2024 at the Ankaran to be used in further sequencing. Upon arrival at the NIB laboratory, gill tissue was dissected immediately and snap-frozen to preserve nucleic acid integrity.

### Seagrass habitat sampling

To identify environmental populations of *Ca.* Thiodiazotropha in the surrounding seagrass habitat, samples for microbial community profiling were collected at both sites from several compartments of the seagrass habitat: seagrass blades, seagrass roots and rhizomes, surrounding sediment, and overlying seawater. Two sampling events were conducted, one in July 2021 and the other in September 2021. During each sampling, five seagrass shoots were chosen haphazardly for sampling. One blade from each shoot was removed using sterile forceps, and a sterile swab was used to sample the microbial communities following the methods of Vogel *et al*. [15]. Pieces of rhizome and the attached roots were collected from five separately chosen shoots. The root and rhizome fragments were detached using sterile forceps and placed into sterile tubes. All blade and root/rhizome samples from the Italian Border site were collected from *C. nodosa*, whereas at the Ankaran site, five of each sample type were collected from *C. nodosa* shoots and five from *Z. noltii.* Additionally, at each site, sediment samples were collected near seagrass roots and placed into sterile tubes. Water column microbial communities were sampled from seawater overlying the seagrass bed by filtering 600 ml through a sterile syringe, and microbial biomass was collected on a 0.22 μM Sterivex filter.

Seagrass blades, seagrass roots and rhizomes, and seawater were immediately fixed in RNAlater, and all samples were placed on ice to be transported to the laboratory at the NIB Marine Biology Station Piran. Samples were kept at −80°C before transport in a dry shipper cooled with liquid nitrogen to the laboratories at the University of Vienna where they were again stored at −80°C until further processing.

During the September 2021 sampling, additional samples were collected to obtain seawater and porewater nutrient concentrations at both sites. For seawater nutrient analysis, samples were collected from the seawater overlying the seagrass bed at each site and filtered through a 0.22 μM filter. Sediment cores were collected at each site, and porewater was extracted from the total length of the core. The collected water samples for nutrient analysis were stored in acid-washed 15 ml tubes and immediately frozen at −20°C. Nutrient analysis (nitrate, nitrite, ammonia, and phosphate) was conducted at the NIB Marine Biology Piran Station using a segmented flow analyzer (QuAAtro, SealAnalytical, [16]). Quality control of analysis was done with certified reference material (CRM) for nutrients in seawater (KANSO TECHNOS) and by annual participation in the intercalibration program (WEPAL-QUASIMEME).

### 16S rRNA gene amplicon analysis

Genomic DNA (gDNA) was extracted from all environmental samples (seagrass blades, seagrass roots and rhizomes, sediment, and seawater) using the DNeasy PowerSoil Pro Kit (Qiagen, Hilden, Germany) following the manufacturer’s instructions. The gill tissue for each *L. orbiculatus* individual was separately homogenized, and gDNA was extracted using the All-In-One DNA/RNA/Protein Miniprep Kit (Bio Basic, Ontario, Canada). All gDNA extracts were transferred to the Joint Microbiome Facility of the Medical University of Vienna and University of Vienna where 16S rRNA gene iTag libraries were prepared for all samples using the archaeal and bacterial primers 515F and 806R (targets the V4 region of *Escherichia coli*) modified by Apprill *et al*. [17] and Parada *et al*. [18]. Amplicons were then sequenced using the MiSeq System (Illumina) in 250 × 250 bp mode, and the raw sequences were processed and joined with DADA2 following the protocols described in Pjevac *et al*. [19]. The constructed amplicon sequence variant (ASV) table was then filtered to remove any sequences resulting from mitochondrial, chloroplast, or eukaryotic DNA. Taxonomy was assigned using the SINA classifier with SILVA reference database v.138. Samples with poor sequence quality (<1000 read pairs), including 13 root/rhizome samples and one gill sample, were excluded from further analysis. The resulting ASV table was then normalized using cumulative sum scaling (CSS) to account for differences in sequencing depth among samples [20]. All statistical analyses were conducted on the CSS-normalized ASV table using R v. 4.2.3 [21].

### Metagenomic analysis

For a subset of the gill samples (86/130), metagenomic sequencing was also performed using short-read sequencing using the MiSeq System (Illumina). Individual read libraries were quality-checked using FastQC v. 0.12.1 [22]. Adapters were trimmed, and phiX contamination was removed using BBDuk (part of BBMap v. 39.10) [23] and reads shorter than 50 bp after trimming were discarded. Reads were k-trimmed from the right with a kmer of 21, minimum kmer of 11 and hamming distance of two along with the “tpe” and “tbo” options. Quality trimming was performed from the right with a Q-score of 15. BBMap’s reformat.sh was used to interleave read libraries, and libraries were merged. Phyloflash v. 3.4.2 [24] was used to generate 16 and 18 small subunit (SSU) rRNA sequences using the SIVLA v. 138.1 database. The interleaved library was assembled using SPAdes v. 4.1.0 [25] in “meta” mode, and BBMap’s reformat.sh was used to remove contigs under 1000 bp from assembly. Prodigal v. 2.6.2 [26] in “meta” mode was used to extract all genes from the assemblies.

To identify candidate host mitochondrial sequences, metagenomic reads were aligned to a custom reference database comprising complete mitochondrial genomes and cytochrome b gene sequences from Lucinidae species using Bowtie2 v. 2.5.4 [27]. Matching reads were deduplicated using fastp v. 0.25.0 [28] and subsequently assembled *de novo* into contigs using SPAdes in single-end mode. Contigs shorter than 1000 bp were excluded from further analysis. The remaining contigs were remapped to the reference database using minimap2 v. 2.30 [29], and the aligned regions were extracted using bedtools v. 2.31.1 [30]. For each sample, the mapped regions were concatenated into a single representative mitochondrial sequence. All sequences were aligned using MAFFT v. 7.526 [31], and phylogenetic trees were inferred using FastTree v. 2.1.11 [32].

To obtain sufficient material for Oxford Nanopore Technologies (ONT) sequencing, gill tissue from seven *L. orbiculatus* individuals collected in March 2024 was pooled. Total DNA was extracted using the ZymoBIOMICS DNA/RNA Miniprep Kit (Zymo Research) following the manufacturer’s instructions. To enrich for high-molecular-weight DNA, an additional clean-up step was performed. Extracted DNA (3–10 μg) was diluted in 60–200 μl of TE buffer (10 mM Tris–HCl, 1 mM Ethylenediaminetetraacetic acid (EDTA), pH 8) and mixed with an equal volume of 2× selective precipitation buffer (2.5% w/v PVP 360000, 1.2 M NaCl, 20 mM Tris-HCl, pH 8). Samples were then centrifuged at 10 000 × g for 30 min at room temperature to pellet fragments >10 kb. The DNA pellet was washed twice with 70% ethanol and resuspended in 50 μl TE buffer. Samples were then incubated at 37°C for 30 min with gentle agitation to facilitate resuspension. The extracted gDNA was then transferred to the Joint Microbiome Facility where it was sequenced using ONT.

### Assembly annotation, mapping, coverage profiling, and differential gene presence

An ONT metagenome-assembled bin representing *Ca*. Thiodiazotropha luna (5 079 676 nt) was used as the reference for downstream analyses. Gene prediction and functional feature annotation of the ONT bin were performed with Prokka v. 1.14.5 [33]. For each sample, short reads were aligned to the ONT-bin reference using Bowtie2, then processed with samtools v. 1.21 to generate sorted, indexed binary alignment map (BAM) files and mapping summaries [34]. Per-gene coverage metrics were computed from BAM files using bedtools to estimate breadth (fraction of each gene covered) and mean depth across each gene interval. To enable cross-sample comparison, gene mean depth was normalized by each sample’s genome-wide mean depth estimated from read-depth profiles, producing normalized depth values. Functional annotation of selected gene sets was performed with eggNOG-mapper v. 2.1.13 [35]. Data aggregation and visualization were performed in Python v. 3.13.5 using pandas/numpy/matplotlib [36–39].

Samples were assigned to symbiont groups based on the dominant type (*Ca.* T. lotti vs *Ca.* T. luna). Each gene in each sample was classified as present, absent, or unknown based on breadth and depth. Genes were classified as present if breadth ≥65% and mean depth met group-specific thresholds (≥20× for luna-dominant samples; ≥2× for lotti-dominant samples). Genes were classified as absent when breadth and mean depth were below predefined cutoffs. All remaining gene–sample combinations were labeled unknown and excluded from testing. For each gene, present vs absent counts were compared between groups using a two-sided Fisher’s exact test, and multiple-testing correction across genes was performed using the Benjamini–Hochberg false discovery rate (FDR) procedure. Circular genome visualization was generated with Circos v. 0.69.10 [40].

## Results

### *L. orbiculatus* gill microbial communities

**Figure 2:**
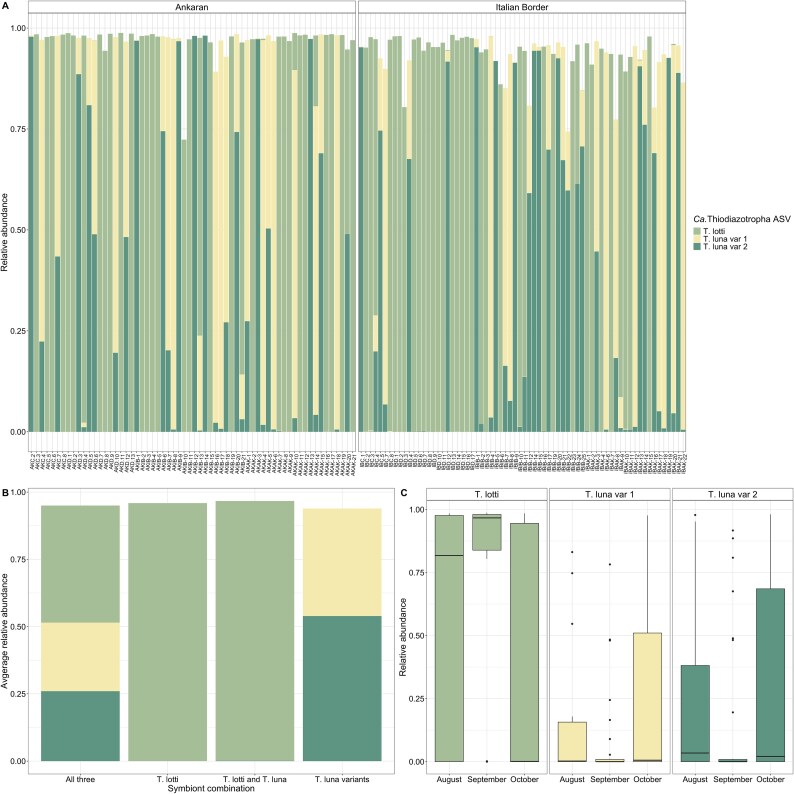
Composition and occurrence of ASVs belonging to *Ca.* Thiodiazotropha in the gill tissue of *L. orbiculatus*. (A) Relative abundances of the three dominant symbiont sequence types in all lucinids (*n* = 130) separated by site, (B) co-occurrence patterns of the three symbiont sequence types, and (C) relative abundances of the three dominant symbiont sequence types by sampling date.

Microbial communities from the homogenized gill tissues of 130 lucinid individuals contained a total of 164 ASVs and had an average richness of 31.8 ASVs. These communities were mainly comprised of *Ca.* Thiodiazotropha spp., the known symbionts of *L. orbiculatus*, with 20 ASVs belonging to this genus accounting for 95% of the relative abundance on average in all individuals. The number of *Ca.* Thiodiazotropha ASVs in each lucinid bivalve ranged from 4 to 13 with a mean of 7.7 ASVs observed. Additionally, there were three *Ca.* Thiodiazotropha ASVs (ASV_7xh_i9e, ASV_imh_tqk, and ASV_ey3_jh5) made up most of this fraction, with at least one of these ASVs comprising the largest percent of relative abundance in each lucinid individual (Fig. 2A). Both the sequence from ASV_7xh_i9e and from ASV_imh_tqk had a 100% match to the 16S rRNA gene region of a species (*Ca.* T. lotti and *Ca.* T. luna, respectively) from metagenome-assembled genomes (MAGs) previously sampled from lucinid gills [5, 14]. The third ASV found in high abundance (ASV_ey3_jhf) also had a very high sequence similarity to *Ca.* T. luna and ASV_imh_tqk with a single base pair difference between the two ASVs.

**Figure 3:**
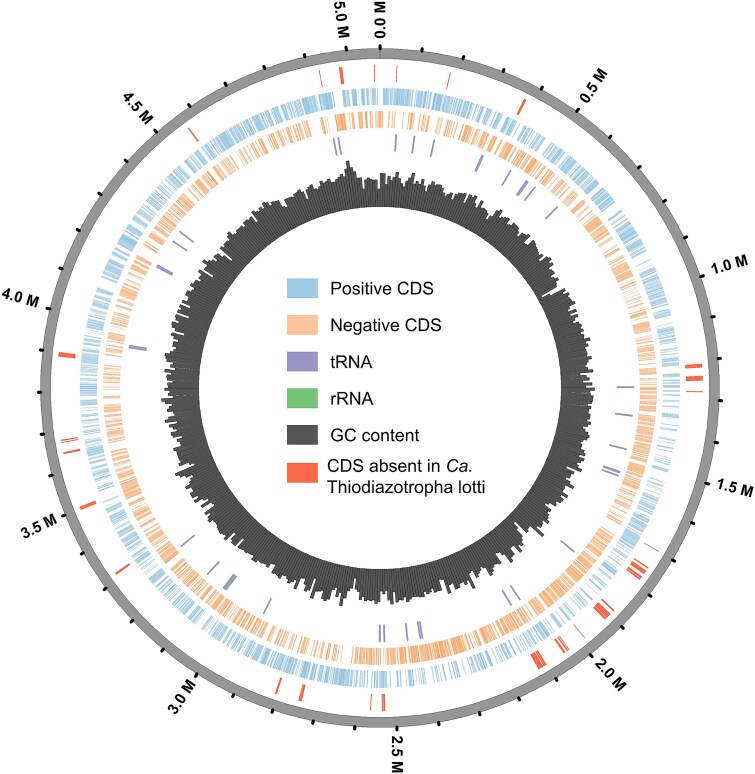
Circular genome map of *Ca.* Thiodiazotropha luna (ONT bin assembly). The assembly is displayed as a circular ideogram with genomic coordinates (Mb) around the perimeter. Coding sequences (CDSs) on the forward (positive) and reverse (negative) strands are shown as bars in the inner two rings, respectively. Transfer RNA (tRNA) genes and ribosomal RNA (rRNA) loci are indicated by bars on the inside of the CDS rings. The inner histogram shows GC content variation across the assembly. Segments on the outer ring highlight the 123 CDS regions classified as present in *Ca.* T. luna but absent in *Ca.* T. lotti (Fisher’s exact test, *P*-value <.05).

To obtain MAGs specific to these very similar ASVs in order to compare genomes, metagenomes from individuals in which one of the ASVs comprised ~97% or more of the 16S rRNA gene amplicon library were selected. These MAGs showed only 96.5% ANI similarity between these two symbiont sequence types. Therefore, these ASVs likely represent “variants” of one another and will be referred to as *Ca.* T. luna variant 1 (ASV_imh_tqk) and *Ca.* T. luna variant 2 (ASV_ey3_jhf). MAG comparisons also confirmed the identities of *Ca.* T. lotti in samples and showed a distinct difference between *Ca.* T. lotti and both *Ca.* T. luna variants with a 78.3%–79.9% ANI similarity. This supports previous findings that *Ca.* T. luna and *Ca.* T. lotti, although closely related, are phylogenetically distinct and more similar to other species of *Ca.* Thiodiazotropha than to one another [14]. To further investigate differences between *Ca.* T. lotti and the *Ca.* T. luna variants, the short reads generated from metagenomic sequencing were compared to the ONT assembled genome for *Ca.* T. luna. Based on this analysis, 123 coding sequence (CDS) regions were likely to be present in *Ca.* T. luna, but absent in *Ca.* T. lotti (Fisher’s exact test, FDR-adjusted *P*-value < .05, Fig. 3, Supplement 1). These regions are associated with a range of cellular functions, including polysaccharide synthesis, cell adhesion, beta-lactamase activity, type 1 restriction modification system, and clustered regularly interspaced short palindromic repeats (CRISPR)-associated proteins.

We investigated the relationship between host mitochondrial variation and symbiont composition using both complete mitochondrial genomes and the *cytochrome b* gene regions. After excluding samples with assemblies shorter than 1000 bp, 34 high-quality samples remained for phylogenetic analysis. Across these samples, all mitochondrial and *cytochrome b* sequences were consistent with those of *L. orbiculatus,* confirming the species identity. To test whether host genotype (i.e. mitochondrial phylogeny) was associated with symbiont community structure, we examined both the dominant symbiont taxon and overall symbiont diversity. Mantel tests showed no significant correlations between host phylogenetic distances and either symbiont richness (*r* ≈ 0, *P* > .05) or relative abundance profiles. Similarly, Pagel’s λ tests for phylogenetic signal indicated no significant association between host phylogeny and the distribution of dominant *Ca.* Thiodiazotropha ASV (λ ≈ 0, *P* > .05), suggesting that host genotype does not constrain symbiont identity or diversity in this system.

In the 130 *L. orbiculatus* individuals sampled, *Ca.* T. lotti comprised the largest fraction of relative abundance in 67 individuals, whereas *Ca.* T. luna variant 2 comprised the majority in 35 and *Ca.* T. luna variant 1 in 28 individuals. The three dominant symbiont sequence types exhibited complex co-occurrence patterns indicating distinct interactions between them (Fig. 2A). *Candidatus* T. lotti occurred alone in 23.8% of lucinid hosts and is the only one of the three dominant symbiont sequence types that occurred alone without a detectable amount of one of the two others. In a small proportion of individuals (14.6%), one of the *Ca.* T. luna variants were observed at a low relative abundance (0.006%–1.9%) with *Ca.* T. lotti comprising the majority and in around 25% of the host individuals, all three dominant symbiont sequence types occurred. In the remaining 36.9% of hosts, the *Ca.* T. luna variants occurred with one another in varying proportions; however, neither of the two variants was ever detected without the other (Fig. 2B). The dominant *Ca.* Thiodiazotropha ASVs comprised an average of 87.9% ± 13.4% SD of the total microbial relative abundance in each bivalve. In host individuals in which the symbiont type comprised the largest fraction, *Ca.* T. lotti ranged from 68.3% to 98.7% relative abundance, whereas the relative abundance of the *Ca.* T. luna variants ranged from 48.4% to 98.1%. The lower range of *Ca.* T. luna variants can be explained by their occurrence always together.

Shell size of bivalves collected in October (*n* = 86) was measured and was significantly different by site with individuals from Ankaran having a larger shell size on average (11.74 ± 2.32 mm, SD) than individuals from the Italian Border (6.15 ± 0.65 mm, SD, Kruskal–Wallis, Χ^2^ = 61.945, df = 1, *P-*value < .0001). However, the dominant symbiont sequence type and the relative abundance of the dominant symbionts did not significantly differ with shell size (Kruskal–Wallis, Χ^2^ = 84.444, df = 83, *P*-value > 0.4, Supplement 2). Further, in lucinids collected from all sampling dates, no significant differences in dominant symbiont sequence type between sites were detected (Kruskal–Wallis, Χ^2^ = 0.083, df = 1, *P*-value = .7769). The relative abundance of the *Ca.* T. luna variants between sites was not significantly different (Kruskal–Wallis, Χ^2^ = 0.019, *P-*value *>* .05, Fig. 2C, Supplement 2) and the relative abundance of *Ca.* T. lotti was marginally significant between sites (Kruskal–Wallis, Χ^2^ = 2.889, df = 1, *P*-value = .089). However, there was a significant difference in the dominant symbiont sequence type among sampling dates with significantly greater relative abundances of *Ca.* T. lotti observed in individuals collected in September than in October (Kruskal–Wallis, Χ^2^ = 15.747, df = 2, *P*-value < .001, Dunn’s test with Benjamini–Hochberg-adjusted *P*-value < .001). Conversely, greater relative abundances of *Ca.* T. luna variant 1 and *Ca.* T. luna variant 2 (Kruskal–Wallis, Χ^2^ = 9.060, df = 2, *P*-value < .05, Dunn’s test with Benjamini–Hochberg-adjusted *P*-value < 0.05, Fig. 2C, Supplement 2) were observed in individuals collected in October than in September. These changes in the relative abundance of the dominant symbiont sequence types over time could be driven by seasonal changes in environmental conditions; however, this dataset is limited by environmental parameters measured only in September.

In September, the Italian Border site had significantly higher concentrations of nitrate (Kruskal–Wallis, Χ^2^ = 7.0313, df = 1, *P-*value = .008) and ammonium in the overlying seawater (Kruskal–Wallis, Χ^2^ = 6.8598, df = 1, *P*-value = .008), as well as significantly higher sediment porewater concentrations of ammonium (Kruskal–Wallis, Χ^2^ = 4.5, df = 1, *P*-value = .034) and phosphate (Kruskal–Wallis, Χ^2^ = 4.6667, df = 1, *P*-value = .031, Table 1, Fig. S1). During the September sampling, *Ca.* T. lotti comprised the largest fraction of relative abundance in more bivalve individuals from the Italian Border (88%) compared to individuals from Ankaran (62%). Additionally, at the Italian Border, *Ca.* T. luna variant 1 was never the dominant symbiont sequence type; yet, at Ankaran, *Ca.* T. luna variant 1 was dominant in 15% of bivalves. However, the dominant symbiont sequence type and relative abundances of the symbiont sequence types did not significantly differ by site in September (Kruskal–Wallis, Χ^2^ = 0.035, df = 1, *P*-value > .05, Supplement 2). This suggests that the site-specific variables measured here, including nutrient concentrations or seagrass species present, are not major determinants of which *Ca.* Thiodiazotropha symbiont sequence type comprises the majority of the symbiont community in individual lucinids.

**Table 1:** Water column and sediment porewater nutrient concentrations at the Ankaran and Italian Border sites.

	**NH4 (μmol/l)**	**NO2 (μmol/l)**	**NO3 (μmol/l)**	**PO4 (μmol/l)**
**Ankaran Seawater (*n* = 5)**	0.04 ± 0.02	0.02 ± 0.005	0.06 ± 0.01	0.01 ± 0.002
**Italian Border Seawater (*n* = 5)**	0.19 ± 0.04	0.02 ± 0.005	0.18 ± 0.03	0.02 ± 0.004
**Ankaran Porewater (*n* = 3)**	15.01 ± 3.33	0.08 ± 0.05	0.69 ± 0.41	1.52 ± 0.072
**Italian Border Porewater (*n* = 4)**	60.53 ± 45.32	0.06 ± 0.02	2.94 ± 1.73	1.88 ± 0.022

Values are mean ± standard deviation.

**Figure 4:**
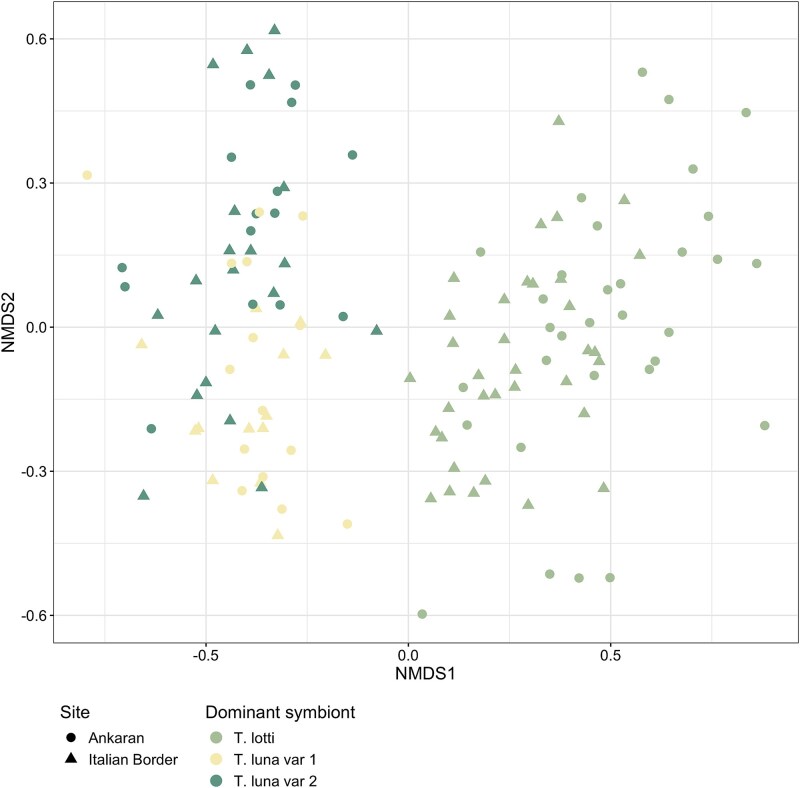
Nonmetric multi-dimensional scaling ordination of the gill microbial communities without the three dominant symbiont sequence types (*Ca.* T. lotti, *Ca.* T. luna var 1, *Ca.* T. luna var 2) showing composition is significantly different by dominant symbiont sequence type even when they are excluded from the species abundance matrix (Bray–Curtis distance, *k* = 3, stress = 0.1695798).

The nondominant remaining proportion of *Ca.* Thiodiazotropha ASVs comprised 0.17%–18.4% in combined relative abundance. As expected, compositional differences in the entire gill microbial community were best explained by the dominant symbiont sequence type due to their high relative abundance and low community evenness (PERMANOVA, *R*^2^ = 0.76, F = 206.45, *P*-value = .001, Fig. S2). When the dominant symbiont sequence types were omitted from the analysis, the remaining gill community still showed significant differences in composition due to the dominant symbiont sequence type, as well as sampling date and marginally significant differences due to site (PERMANOVA, *P*-value = .001, *P-*value = .005, *P-*value = .071, Supplement 2) with distinct clustering of those communities dominated by *Ca.* T. lotti and dominated by the two *Ca.* T. luna variants remained (Fig. 4). This suggests that the dominant symbiont sequence type may be interacting with the bivalve host or modifying the gill microenvironment to shape the overall gill microbial community.

### *Ca.* Thiodiazotropha spp. in the surrounding seagrass habitat

**Figure 5:**
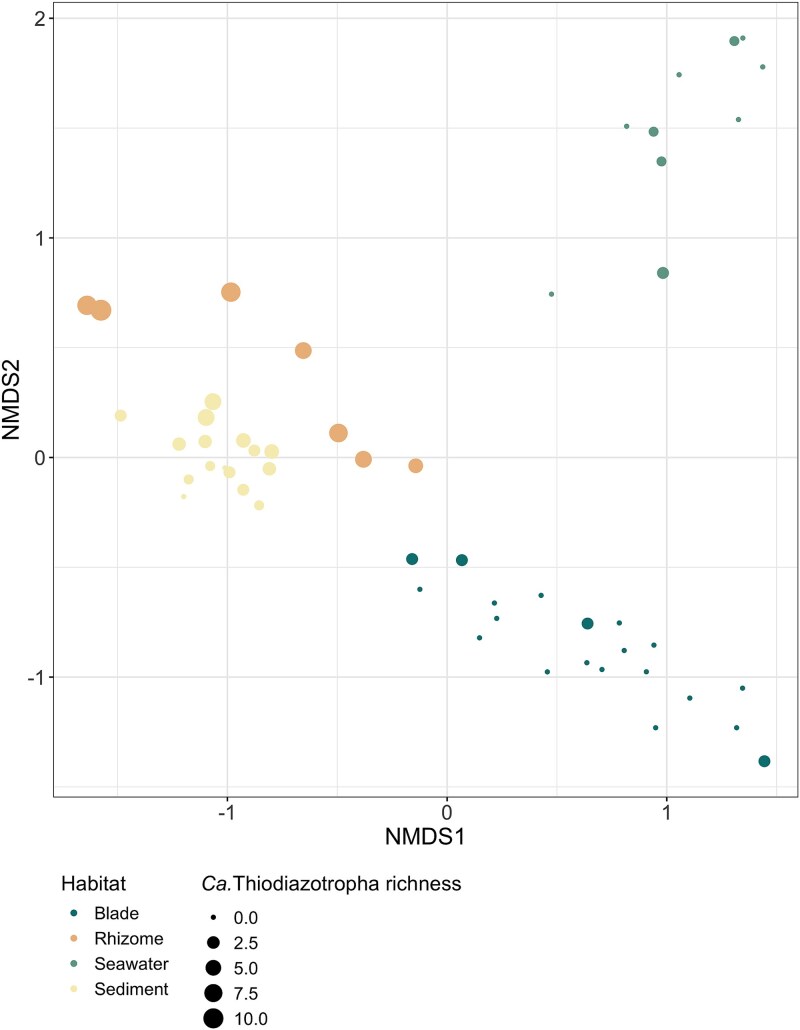
Nonmetric multi-dimensional scaling ordination of microbial communities from the seagrass habitat. Colors indicate the habitat compartment and size of the point indicates the observed richness of *Ca.* Thiodiazotropha ASVs in the community (Bray–Curtis distance, *k* = 3, stress = 0.06764439).

**Figure 6:**
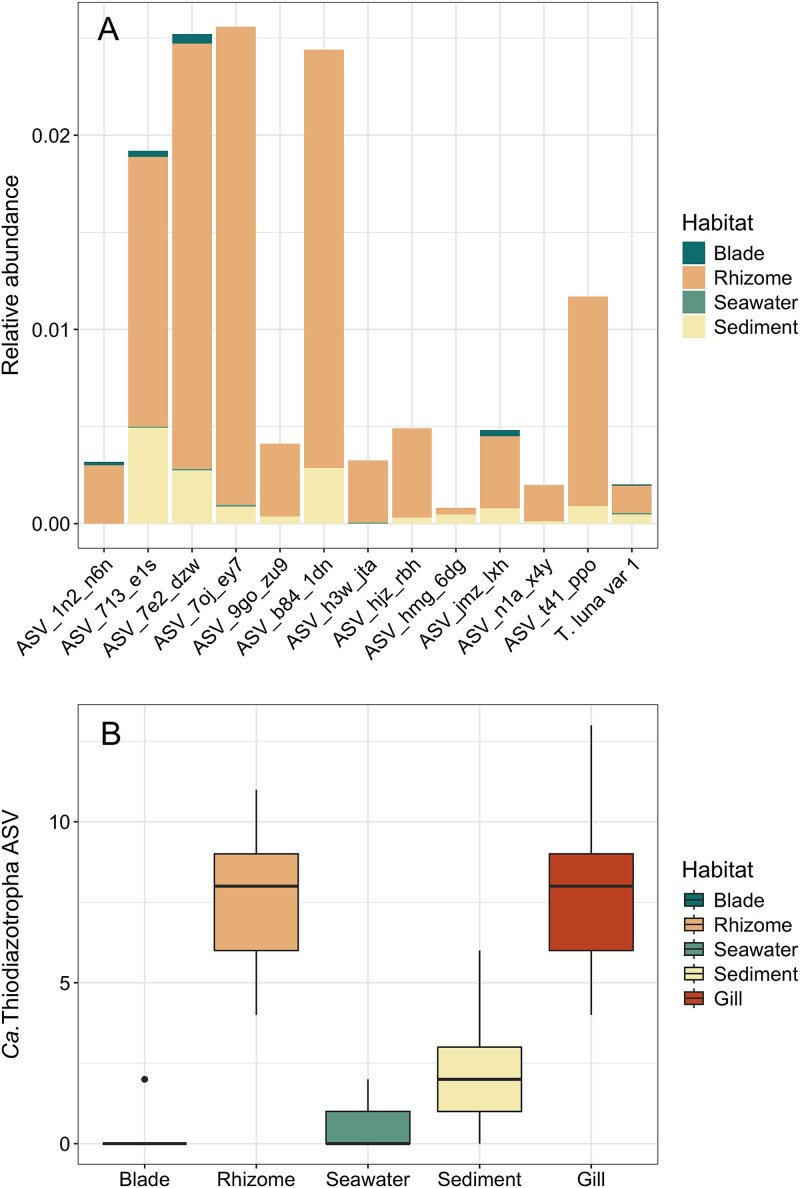
Composition of ASVs belonging to *Ca.* Thiodiazotropha detected in the environmental reservoir. (A) Relative abundance of *Ca.* Thiodiazotropha ASVs in the four compartments of the seagrass habitat showing significantly greater abundance in the seagrass rhizome and (B) richness of *Ca.* Thiodiazotropha ASVs in the seagrass habitat and lucinid gills.

**Figure 7:**
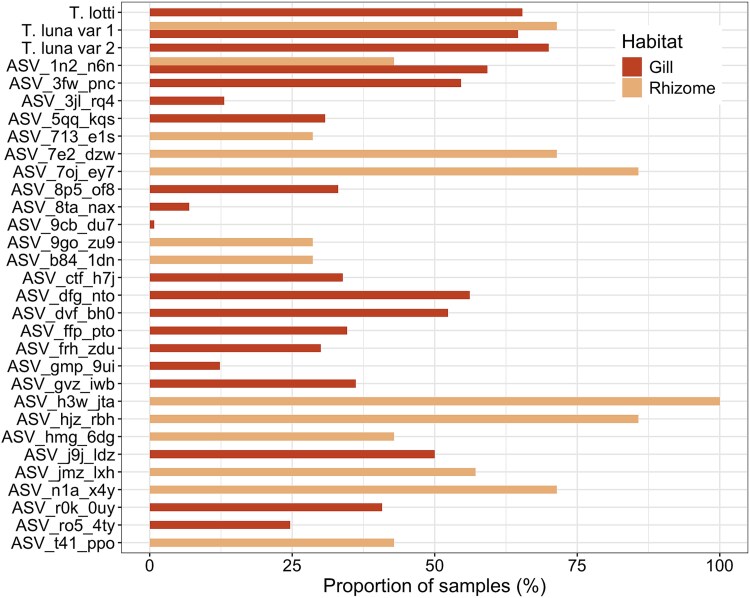
Occurrence of all *Ca.* Thiodiazotropha ASVs observed in the gill and rhizome with *Ca.* T. Luna variant 1 and ASV_1n2_n6n occurring in both habitat types.

Microbial communities from the different compartments of the seagrass habitat (seagrass blade, seagrass root and rhizome, seawater and sediments) had a distinct composition and structure (PERMANOVA, *R*^2^ = 0.46, *P*-value = .001, Fig. 5). Although ASVs belonging to *Ca.* Thiodiazotropha were found in all compartments of the environment at low abundances, the root and rhizome had significantly higher relative abundances of *Ca.* Thiodiazotropha ASVs than all other sample types (Kruskal–Wallis, Χ^2^ = 31.689, df = 3, *P*-value < 0.001, Dunn’s test with Benjamini–Hochberg-adjusted *P*-value < .05, Fig. 6A). The microbial communities on the roots and rhizomes also had the highest *Ca.* Thiodiazotropha richness with 11 of the 13 ASVs found in the seagrass environment observed on the roots and rhizomes (Fig. 6B). The higher richness and abundances of *Ca.* Thiodiazotropha ASVs observed on the roots and rhizomes are consistent with previous findings that this genus of sulfur-oxidizing bacteria is a consistent member and enriched in the seagrass rhizosphere and possibly engages in a beneficial symbiosis with the seagrass host [8]. Two of the *Ca.* Thiodiazotropha ASVs (*Ca.* T. luna variant 1 and ASV_1n2_n6n) found in the seagrass habitat were also detected in the gill tissue (Fig. 7). *Candidatus* T. luna variant 1 was detected in all compartments of the seagrass habitat, whereas ASV_1n2_n6n was only detected on the seagrass blades and rhizome. ASV_1n2_n6n occurred in ~60% of all lucinid individuals at a relative abundance of <1% with the exception of two individuals in which it occurred at 4.4% and 17.9%. Additionally, it was found at a slightly higher relative abundance in the rhizome (~0.04%) than *Ca.* T. luna variant 1 (~0.02%), although *Ca.* T. luna variant 1 occurred more frequently in a greater number of samples. Additionally, many of the other *Ca.* Thiodiazotropha ASVs that were not observed in the lucinid gill were more prevalent on the seagrass root/rhizome than the two shared ASVs (Fig. 6A). Yet, this shows that *Ca.* T. luna variant 1 and ASV_1n2_n6n can utilize both the seagrass and lucinid bivalves as a host and suggests that symbiont sharing may be occurring within the seagrass habitats.

## Discussion

This study showed that a greater *Ca.* Thiodiazotropha diversity occurs in *L. orbiculatus* than previously observed with three dominant symbiont sequence types commonly co-occurring in the population and within a single individual. Further, a new symbiont sequence type, *Ca.* T. luna variant 2, was identified and was observed to occur frequently and sometimes at high abundance in individual bivalves at the two sites. A previous study [12] observed similar fine-scale diversity patterns in the lucinid bivalve *Ctena orbiculata* with four coexisting *Ca.* Thiodiazotropha-like symbiont sequence types in the population. The three dominant symbiont types in this study exhibited complex co-occurrence patterns and varied in their relative abundance within individual hosts. Although it is known that *Ca.* Thiodiazotropha colonizes the lucinid host during early developmental stages, whether symbiont uptake is continued throughout the lifetime of the lucinid host and the mechanism of colonization need to be resolved to better interpret these patterns of diversity.

The observed relative abundances of the three dominant *Ca.* Thiodiazotropha symbionts in this study did not differ with respect to the two sites despite the sites having differences in porewater and water column nutrient concentrations, shell size, and seagrass species present at the site. However, the dominant symbiont sequence type did differ by sampling date, which could be driven by environmental variables not measured or by changes to gill morphology due to the reproductive cycle. In *L. orbiculatus,* the ratio of bacteriocytes to mucocytes can decrease during warm periods, which could affect patterns of symbiont relative abundance, and is thought to coincide with spawning periods [41]. Further, the observed changes in abundance of the dominant symbiont types over time could indicate symbiont turnover within the host.

The *Ca.* Thiodiazotropha symbiont types in our study differed in genes relating to cellular function, which could explain how multiple, closely related symbiont types have the ability to colonize the same host species, underlying the symbiont diversity observed. However, none of the genes likely to be present in *Ca.* T. luna and absent in *Ca.* T. lotti were associated with metabolism. A closed genome of *Ca.* T. lotti is needed to do the reciprocal analysis and confirm that these symbiont types do not have differences in metabolic capability, including ability to fix nitrogen. This differs from previous studies that have shown distinct differences between chemosynthetic symbionts that coexist in the same host individual, including the two *Ca.* Thiodiazotropha spp. that were observed together in the lucinid *C. costata* [5, 42]*.* However, results also show that the dominant symbiont sequence type present in a lucinid individual may structure the remaining bacterial community on and within the gill. This suggests that the dominant symbiont sequence types may be interacting differently with their bivalve host, modifying the gill microenvironment or directly interacting with co-occurring microbes in the gill. In the future, experimental data such as metatranscriptomes from *L. orbiculatus* hosting different symbiont sequence types could shed light on phenotypic differences.

As *Ca.* Thiodiazotropha symbionts are horizontally transferred from the parent to the offspring through the environment by *L. orbiculatus* and other lucinid species [11, 43, 44], it has long been assumed that these symbionts must have an environmental reservoir. Although many ASVs belonging to *Ca.* Thiodiazotropha were observed in the seagrass habitat, none were observed in high relative abundance, in agreement with previous studies [8]. Further, only two of the *Ca.* Thiodiazotropha ASVs associated with *L. orbiculatus* were also detected within the environmental reservoir. The inability to detect all *Ca.* Thiodiazotropha symbiont types in the environment could be limited by sampling depth or abundances of *Ca.* Thiodiazotropha symbionts in the reservoir may vary temporally with spawning events and time of symbiont acquisition.

The *Ca.* Thiodiazotropha ASVs present in the environmental reservoir occurred in significantly higher abundances on the seagrass roots and rhizomes than in the other environmental compartments sampled. These results confirm a previous meta-analysis of seagrasses worldwide [8] and suggest that *Ca.* Thiodiazotropha may be specific and permanent members of the seagrass rhizosphere microbial community. This association could enable seagrasses to mitigate harmful levels of sulfide in sediments with no lucinid bivalves present and may be critical for optimal seagrass growth, particularly considering that the ability to fix nitrogen is common in shallow water *Ca.* Thiodiazotropha spp. and nitrogen is often the limiting nutrient for seagrass growth [9, 45–48]. A similar symbiosis has been demonstrated in the salt marsh grass *Spartina alterniflora* where close relatives of *Ca.* Thiodiazotropha contribute to sulfur oxidation on the roots with increased levels of expression of sulfur-oxidizing genes (oxidative *dsrAB*) on roots under sulfide stress [49]. The extremely close relationship between bivalve- and seagrass-associated *Ca.* Thiodiazotropha, with identical 16S rRNA genes in the region we sequenced, shows that some symbionts in the environmental pool are shared between hosts within the seagrass habitat. However, additional genomic data are needed to show whether truly genetically identical symbionts can colonize both hosts, or if minor genetic changes are required to adapt to association with different hosts, as was previously shown with a single promoter in *Vibrio fischeri* colonizing squid or fish [50].

Lucinid bivalves, their symbionts, and numerous seagrass species are involved in a three-way facilitative relationship resulting from millions of years of shared geologic and evolutionary history. Yet, it is unclear how rapid changes in the environmental conditions and host availability will affect these facilitative interactions and coevolution [51]. Seagrass coverage is declining at an unprecedented rate due to anthropogenic activities, and this loss is often coupled with a decrease in seagrass diversity as species composition shifts from long-lived, climax species to shorter-lived, opportunistic species [52, 53]. The decrease in abundance and loss of biodiversity of the seagrass host could reduce the amount of symbiont sharing and symbiont genetic diversity within the seagrass habitat, thus weakening seagrass–symbiont interactions. However, the association of both bivalves and seagrasses with *Ca.* Thiodiazotropha symbionts may allow these hosts to better mitigate degraded environmental conditions and these facilitative interactions may persist or even strengthen with changing environmental conditions. A previous study showed that the genetic diversity of the chemosynthetic endosymbionts associated with a genus of marine snails (*Alviniconcha*) was due to adaptation of the symbionts to local environmental conditions rather than host genetics [54]. Association of the host organism with locally adapted symbionts may provide the host with fitness advantages, especially under potentially stressful conditions. The amount and directionality of symbiont sharing needs to be determined in order to assess how changes in host availability could affect symbiont diversity and their relationships to both hosts, especially under changing environmental conditions [55–57].

## Conclusion

We showed that two of the *Ca.* Thiodiazotropha ASVs found on the seagrass roots and rhizomes were also found in the gill tissue of *L. orbiculatus* at the same site and time. This reveals not only that seagrass roots may offer a suitable niche for *Ca.* Thiodiazotropha, as well as certain species of *Ca.* Thiodiazotropha may be able to use both seagrass and bivalves as hosts. Symbiont sharing between two different hosts in a shared habitat has important implications for *Ca.* Thiodiazotropha ecology and evolution, and for the interactions between the two hosts. For example, it may support transmission between host generations or maintain a higher diversity of symbionts in both the bivalve and the seagrass hosts. This diversity, in turn, may enable seagrasses to endure a broader range of stressful conditions and promote growth, underpinning their essential ecosystem services. To build upon this, future studies should investigate the functions *Ca.* Thiodiazotropha performs on seagrass roots and rhizomes and how they interact with the seagrass host. A better understanding of how these symbionts move through the environment is also needed to determine the time scale of host switching in these systems and how such closely related or possibly even identical organisms manage to successfully colonize the intracellular environment in animals as well as the surfaces of plant roots. Such intimate and beneficial associates as *Ca.* Thiodiazotropha that colonize host animal gill cells are usually highly specific for their co-evolved host organism (e.g. [58]). Although some bacterial pathogens can associate with hosts across an equally broad taxonomic range (e.g. [59, 60]), this would be an unprecedented ability for beneficial symbionts.

## Supplementary Material

FigureS1_wrag094

FigureS2_wrag094

Supplementary_materials_wrag094

## Data Availability

The 16S rRNA gene amplicon sequencing, metagenomic, and long-read (ONT) data generated in this study have been deposited at the Sequence Read Archive under the BioProject accession PRJNA1378740.
