# Supplementary figures and images for: Symbiont diversity within Loripes orbiculatus and the case for multiple hosts
Source: ISME J. 2026 Apr 15;20(1):wrag094. doi: 10.1093/ismejo/wrag094 (PMC13167027; doi:10.1093/ismejo/wrag094)

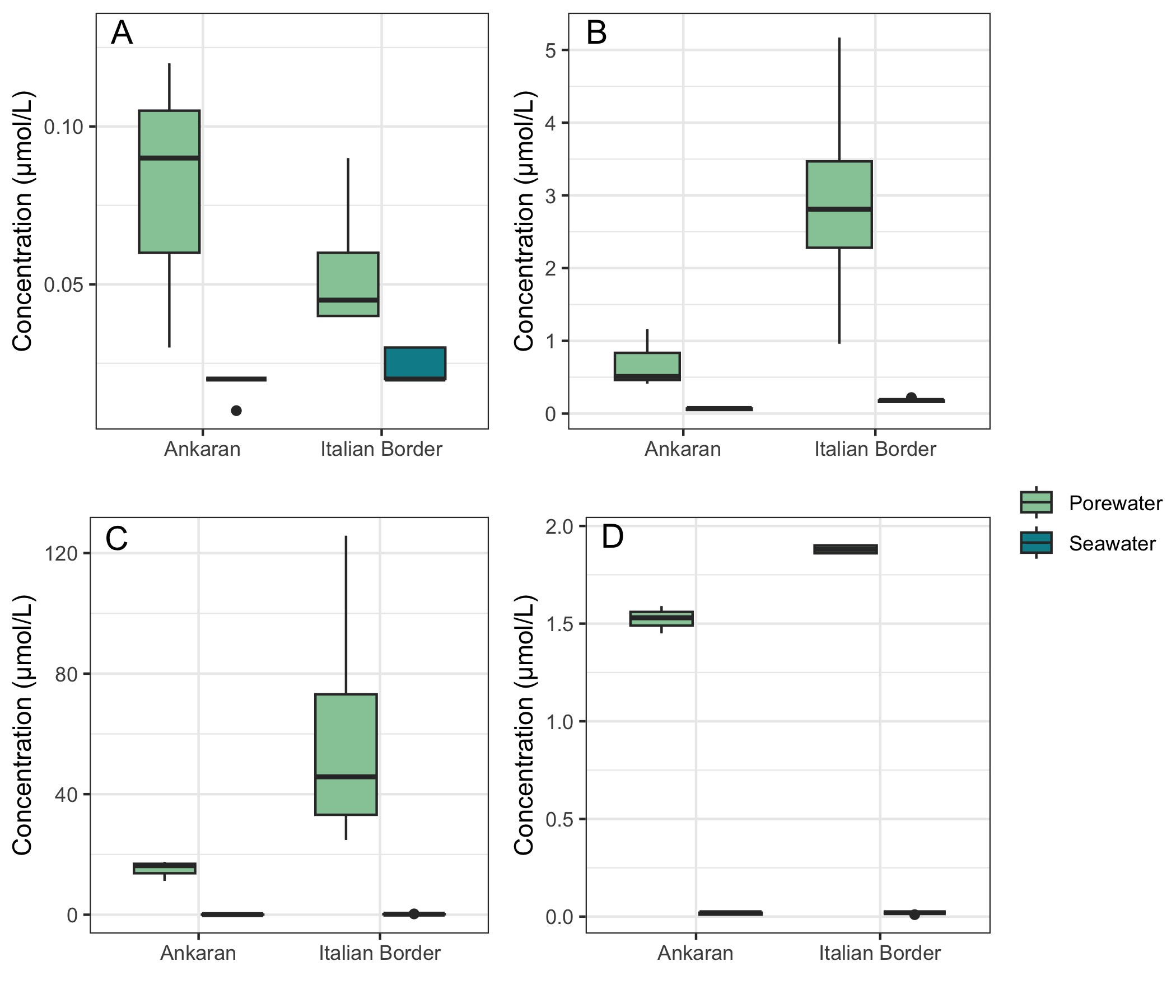

Supplement: FigureS1_wrag094 [file figures1_wrag094.jpeg]

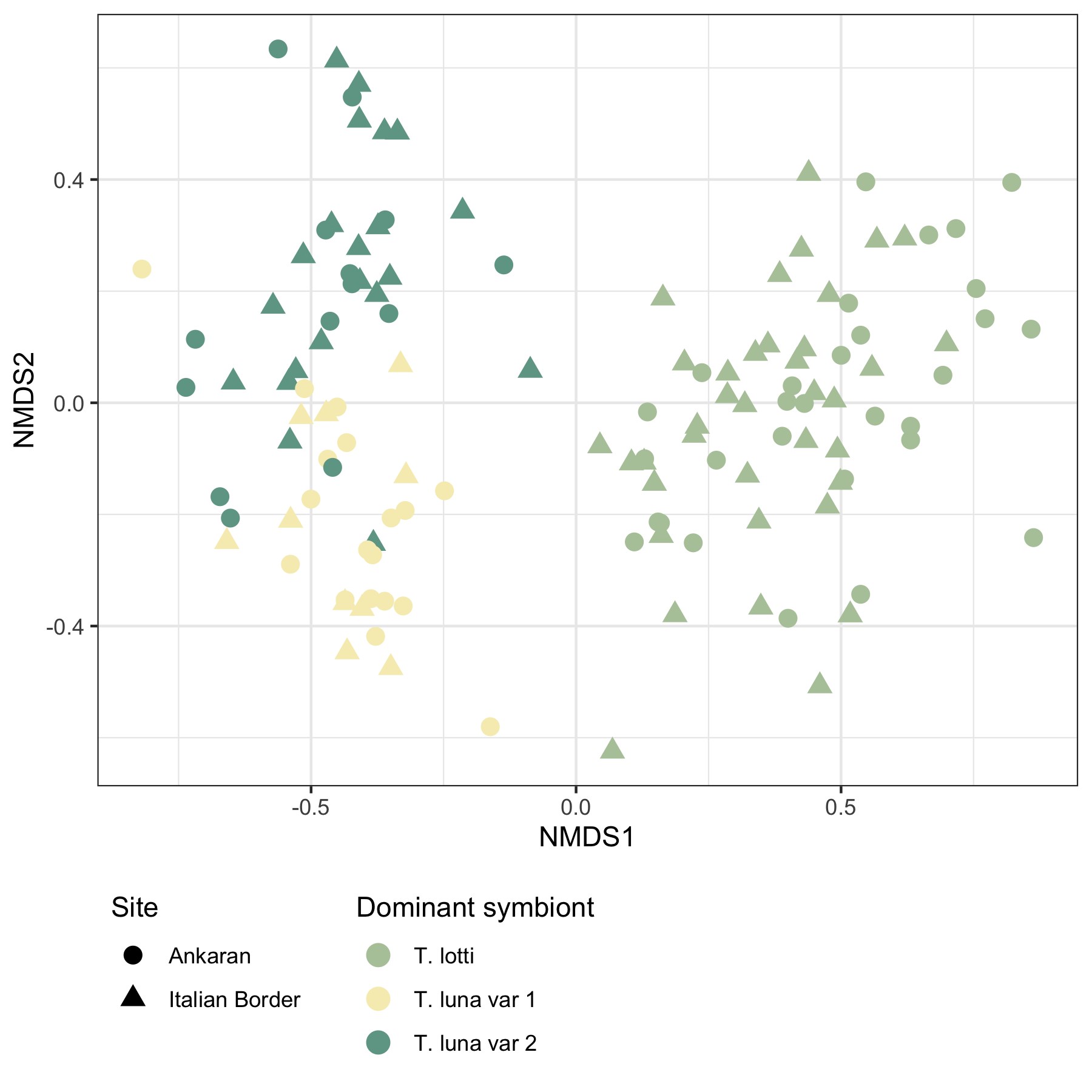

Supplement: FigureS2_wrag094 [file figures2_wrag094.jpeg]
