# Supplementary material for: Symbiont diversity within Loripes orbiculatus and the case for multiple hosts
Source: ISME J. 2026 Apr 15;20(1):wrag094. doi: 10.1093/ismejo/wrag094 (PMC13167027; doi:10.1093/ismejo/wrag094)
Supplement: Supplementary_materials_wrag094 [file supplementary_materials_wrag094.zip › Supplementary Figure Legends.docx]

**Supplementary Figure Legends**

**Figure S1.** Nutrient concentrations in the porewater and overlying seawater at both sites. The measured variables are **(A)** nitrate, **(B)** nitrite, **(C)** ammonium, and **(D)** phosphate.

**Figure S2.** Non-metric Multi-Dimensional Scaling ordination of the gill microbial communities including the three dominant symbiont sequence types (*Ca.* T. lotti, *Ca.* T. luna var 1, *Ca.* T. luna var 2) showing compositional differences due to dominant sequence type (Bray-Curtis distance, k=3, stress=0.11632).
